# Engineering Parameter Window for Filament Formation and Early-Stage Evolution in Embedded Printing of Gelatin/Alginate Hydrogels Within Carbomer Support Baths

**DOI:** 10.3390/gels12080749

**Published:** 2026-08-21

**Authors:** Jinwei Li, Wang Tang, Zihang Yan, Huansi Mo, Jinhu Wang, Lin Lin, Yong Wang, Hui You, Yuanfen Chen

**Affiliations:** School of Mechanical Engineering, Guangxi University, Nanning 530004, China; 2211391025@st.gxu.edu.cn (J.L.); 2411301053@st.gxu.edu.cn (W.T.); 2301180120@st.gxu.edu.cn (Z.Y.); 2301180107@st.gxu.edu.cn (H.M.); 2411301054@st.gxu.edu.cn (J.W.); llin@gxu.edu.cn (L.L.); wangy72@gxu.edu.cn (Y.W.)

**Keywords:** extrusion printing, embedded printing, yield stress fluid, printability, hydrogel, process window

## Abstract

Embedded 3D printing of hydrophilic hydrogels in aqueous support baths often suffers from filament deformation, positional deviation, and diffusion before complete cross-linking, while practical parameter-selection guidelines for specific material systems remain limited. In this work, an integrated engineering evaluation method was developed to characterize filament formation and early-stage evolution during embedded printing of gelatin/sodium alginate inks in Carbomer support baths. Filament quality was assessed using cross-sectional geometry, contour irregularity, deposition position, and early-stage diffusion ratio. The effects of printing kinematics and material rheology are systematically examined to establish a practical process window for this hydrophilic ink–bath system. The results show that the speed ratio between substrate and ink is a primary factor controlling filament geometry and deposition position, and a ratio close to 1 yields the most balanced cross-sectional morphology without position shift from the printed nozzle. Ink with high viscosity maintains contour irregularity within a narrow range of 0.005–0.009 and suppresses early diffusion, whereas a support bath with high viscosity and ink with high viscosity combination further slows diffusional evolution by about 70%. Based on the identified process window, several three-dimensional hydrogel structures were fabricated successfully. The proposed workflow provides a practical route for process evaluation and parameter selection in hydrophilic embedded printing, especially for continuous filament and tubular structures, and offers engineering guidance for bio-soft material fabrication in applications.

## 1. Introduction

Additive manufacturing (AM) has emerged as a powerful platform for fabricating hydrogel-based constructs, encompassing techniques such as extrusion-based bioprinting, inkjet bioprinting, stereolithography (SLA), and laser-assisted bioprinting [1,2]. Each method offers distinct capabilities: extrusion-based printing accommodates high-viscosity bioinks and fits well to clinically relevant dimensions; inkjet printing achieves micron-scale droplet resolution; SLA provides superior feature fidelity through localized photopolymerization; and laser-assisted printing enables gentle, high-precision cell transfer [1,2]. The choice of hydrogel ink is equally important: natural polymers such as gelatin, alginate, hyaluronic acid, and gelatin methacryloyl (GelMA) remain widely adopted owing to their biocompatibility, shear-thinning rheology, and capacity for ionic or photochemical cross-linking [3,4]. Recent studies have further expanded the functional scope of 3D-printed hydrogels, including carbon-dot-infused constructs with antibacterial and mechano-fluorescent sensing capabilities [5] and patient-specific 3D-printed therapeutic devices [6]. Despite these advances, there is a common challenge: many hydrogel inks are too soft to support their own weight during layer-by-layer deposition, limiting the geometric complexity achievable with conventional AM approaches.

Embedded 3D printing is an extrusion-based additive manufacturing strategy in which the printed ink is deposited inside a support bath rather than in air. This approach enables the fabrication of soft and low-modulus materials that are otherwise difficult to print into self-supporting geometries, and has therefore attracted considerable attention in flexible sensors [7,8], soft robots [9,10], tissue engineering [11,12], and biomimetic construct fabrication [13]. In contrast to conventional extrusion printing, embedded printing relies on a support medium with yield stress behavior to temporarily hold the deposited material and reduce the influence of gravity, density mismatch, and filament collapse during deposition [14,15,16,17,18,19]. As a result, the final printing outcome is jointly affected by deposition kinematics, rheological properties of both the ink and the support bath, interfacial interactions between the two phases, and structural evolution of the deposited filament before complete stabilization. Specifically, Hua et al. [20] emphasized that print fidelity depends on coupled factors spanning from the pre- to post-printing stages, and Deng et al. [21] showed that ink rheology, bath properties, and interfacial interactions collectively govern print quality in all-aqueous embedded printing. For aqueous hydrogel systems in particular, these factors are strongly coupled, making process selection largely empirical in many practical cases.

Previous studies on embedded printing can be broadly grouped according to their primary focus. The first group addresses deposition kinematics and filament formation during printing. These studies have shown that extrusion pressure, nozzle size, and the relative matching between nozzle movement speed and ink extrusion speed strongly influence filament expansion, stretching, and apparent diameter [22,23]. Such work provides useful guidance for identifying printable conditions, but it mainly evaluates the filament at the moment of deposition and does not fully account for subsequent morphology changes inside the bath. The second group focuses on rheology-controlled printing stability, showing that yield stress, viscoelasticity, and the rheological contrast between the ink and support bath govern the size of the yielded zone, shape retention, and overall print fidelity [24,25]. These studies are important for understanding bath-assisted extrusion, yet they are often centered on stability criteria or rheological descriptors rather than on a unified engineering workflow for evaluating multiple quality attributes of the printed filament. A third group of studies examines interfacial effects and filament morphology regulation. Numerical and experimental investigations have shown that surface tension and local viscosity ratio can influence filament roundness, smoothness, and interfacial stability, and that excessive interfacial stress may lead to droplet formation or filament breakup [26,27,28,29]. These results clarify how filament morphology depends on ink–bath pairing, but their implications are often discussed from the standpoint of specific mechanisms. A fourth group of studies highlights post-deposition evolution inside the support bath, especially diffusion and delayed stabilization [30]. Lei et al. found that photopolymerized acrylamide ink failed to crosslink in the Carbomer support bath due to diffusion, and that collagen ink printed in Carbomer, gelatin, and agarose support baths showed a deviation of 10–20% between the structural area and the CAD model dimensions [31]. These findings indicate that print quality is not determined solely at the instant of deposition; instead, the short period between deposition and complete stabilization can substantially affect final dimensional accuracy. However, this stage is often discussed separately from in-print filament formation, and integrated evaluations covering both deposition behavior and early in-bath evolution remain limited. For hydrophilic ink–hydrophilic bath systems, this gap is particularly relevant. In such systems, the deposited filament must not only maintain a regular shape during extrusion, but also resist positional drift and diffusional evolution before full cross-linking is completed. Therefore, from an engineering perspective, there is a need for a practical evaluation route that links printing kinematics, material rheology, filament quality, and short-term in-bath stability within the same experimental framework.

**Figure 1:**
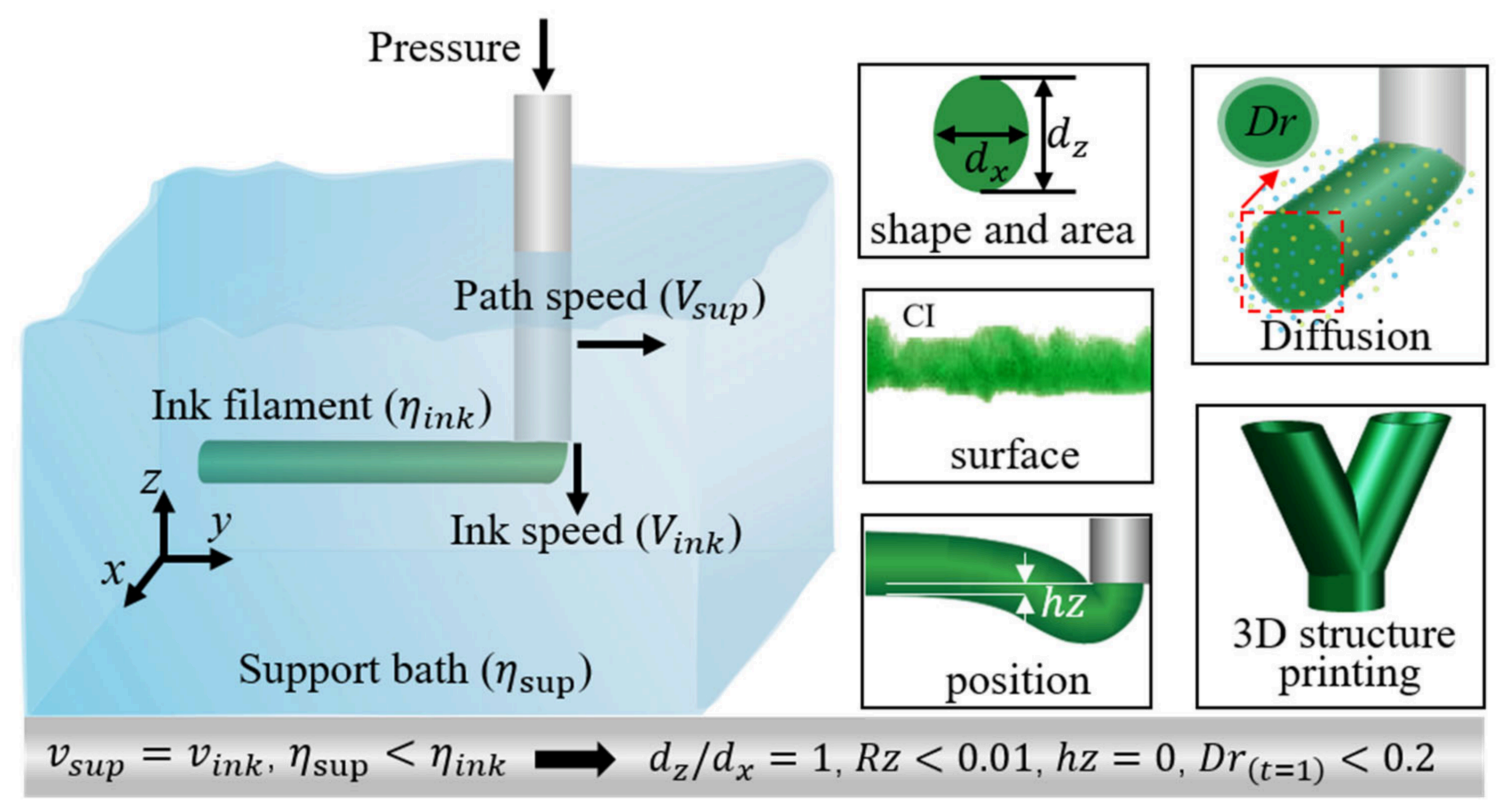
Schematic diagram of the research parameters and characterization indexes. *v_sup_* is the platform moving speed, *v_ink_* is the ink extrusion speed, *η_ink_* is the viscosity of the ink, *η_sup_* is the viscosity of the support bath, *d_z_* is the diameter of the filament in the *z* direction, *d_x_* is the diameter of the filament in the *x* direction, *Rz* is the surface roughness of the printed filament, *hz* is the deposition position of the filament along the *z*-axis relative to the nozzle-tip plane, *CI* is the contour irregularity index, and *Dr* is the diffusion ratio.

In this work, we focus on an engineering-oriented evaluation of filament formation and early-stage evolution in a representative hydrophilic embedded printing system, as illustrated in Figure 1. Carbomer microgels were selected as the support bath and gelatin/sodium alginate was used as the printing ink [32,33]. Rather than proposing a universal physical model for all embedded printing conditions, this study aims to establish a practical parameter-selection route for this specific ink–bath combination by integrating the assessment of deposition morphology and short-term in-bath evolution. Filament quality was evaluated from four aspects: cross-sectional geometry, image-based contour irregularity, deposition position, and early-stage diffusion evolution. The influences of printing speed ratio and rheological properties were then analyzed to identify the dominant factors governing filament regularity, positional accuracy, and short-term stability before secondary cross-linking. Finally, three-dimensional hydrogel structures were printed to verify the engineering applicability of the identified process window. Unlike most studies which only focus on the printing kinetics or the diffusion process, this study shows how the printing parameters and material rheology affect both the printing kinetics and early diffusion process, which affect the final shape of the printed filament. This study is intended to provide experimentally grounded guidance for hydrophilic embedded printing where both immediate filament formation and subsequent in-bath evolution must be considered together.

## 2. Results and Discussion

### 2.1. Rheological Properties of Materials

In this paper, hydrophilic biomaterials were used as the object of study. The support bath was made of Carbomer micro-hydrogel, which is a bio-friendly material and possesses good yield stress and transparency [34,35]. The inks were made from the natural bio-materials sodium alginate and gelatin, which have a variety of biomedical applications [36,37]. Among them, gelatin possesses thermo-solidifying properties, while alginate can chelate with divalent cations to form alginate hydrogel, and the inks composed of them can prepare hydrogels with a double cross-linked network interpenetrating structure.

**Figure 2:**
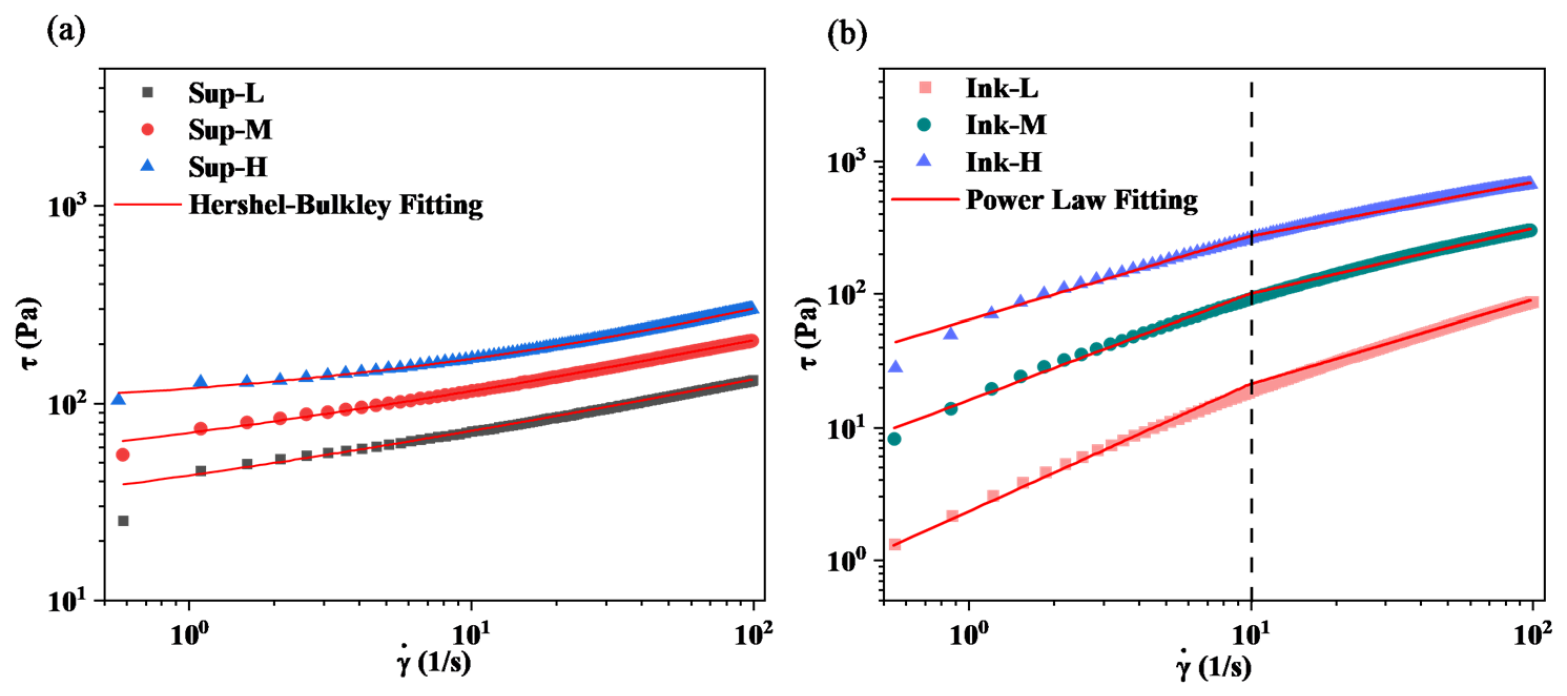
Shear rate versus shear stress curves. (**a**) Carbomer support bath; (**b**) sodium alginate/gelatin ink.

For embedded 3D printing, the rheological properties of the support bath and ink are very important. Three different support baths and inks were prepared respectively, denoted bath/ink-H/M/L according to the shear stress. The shear rate versus shear stress curves of the support baths were obtained by rheological testing, as shown in Figure 2a, where the shear stress increases with the increase in the Carbomer mass fraction at the same shear rate. The Herschel–Bulkley model [38], as shown in Equation (1), is fitted to Figure 2a to obtain the yield stresses of Carbomer with different micro-hydrogel mass fractions, and the resulting coefficients are shown in Table 1. The yield stress of the support bath increased as the mass fraction of the Carbomer increased. Both the ink and the support bath were shear-thinning non-Newtonian fluids, while the Carbomer support bath had a yield stress because of its hydrogel particles. The ink did not have a yield stress, as shown in Figure 2b. According to the shear rate versus shear stress curves obtained from the rheological tests, a piecewise power-law fluid model [39], as shown in Equation (2), is used to fit Figure 2b, and the resulting coefficients higher than 10 are shown in Table 1, and coefficients lower than 10 are shown in Table A1. The values would be higher than 10 during the printing process, which we explain in more detail in the following sections. The shear stress of the inks increased with increasing mass fraction of sodium alginate (gelatin mass fraction was fixed). The shear stress of Ink-L was smaller than those of the three support baths (at the same shear rate), and the shear stress of Ink-H was larger than those of the three support baths, indicating that among the support baths and inks, the one with the strongest ability to resist shear deformation was Ink-H, and the weakest one was Ink-L.

(1) $$\sigma =\sigma_{0}+K{\dot{\gamma }}^{n}$$

where *σ* is the shear stress, *σ_0_* is the yield stress of the support bath, *K* is the consistency coefficient, $\dot{\gamma }$ is the shear rate, and *n* is the flow behavior index.

(2) $$\sigma =K{\dot{\gamma }}^{n}$$

where *σ* is the shear stress, K is the consistency coefficient, $\dot{\gamma }$ is the shear rate, and *n* is the flow behavior index.

### 2.2. Characterization of Printability

#### 2.2.1. Volumetric Morphology

**Figure 3:**
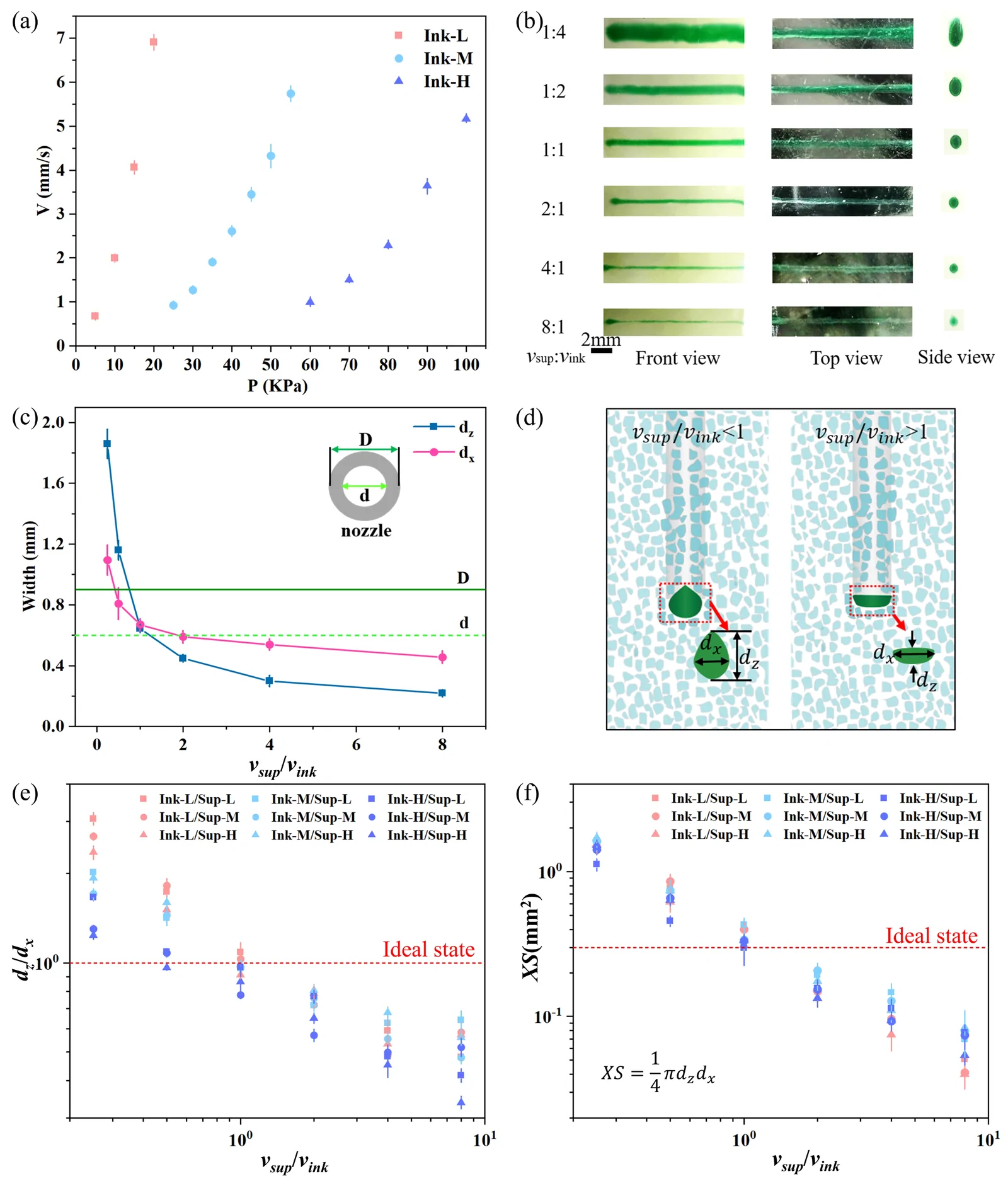
(**a**) Applied print air pressure versus ink extrusion speed; (**b**) processed images of printed filaments projected in three directions: front view, top view, and side view at different speed ratios (Sup-H and Ink-H, *v_ink_* = 2.14 mm/s); (**c**) variation curves of *d_z_* versus *d_x_* at different speed ratios (Sup-M and Ink-M; *v_ink_* = 2.14 mm/s; D = 0.9 mm is the outer diameter of the nozzle; d = 0.6 mm is the inner diameter of the nozzle); (**d**) schematic of the relationship between *d_z_* and *d_x_* at different speed ratios; (**e**) curve of filament diameter ratio *d_z_/d_x_* versus *v_sup_/v_ink_* (*v_ink_* = 2 mm/s); (**f**) curve of cross-sectional area *XS* versus *v_sup_/v_ink_* (*v_ink_* = 2 mm/s). Here, *XS* is the average cross-sectional area of the filament calculated from the mean of *d_z_* and *d_x_*, and *v* is the printing speed.

The applicable air pressure for different ink extrusion printing was determined by weighing method, and their corresponding mass flow rates *Q_m_* were obtained. The extrusion speeds corresponding to the printing air pressure for different inks were obtained from *Q_m_*. As shown in Figure 3a, the printing flow rate of different inks increased in a pressure-dependent manner with the increase in air pressure, and this phenomenon was consistent with the characteristic of the shear thinning of the inks. As the mass fractions of sodium alginate increased from Ink-L to Ink-M to Ink-H, their apparent viscosity increased and the ink with higher viscosity had more resistance in the extrusion process, and required higher air pressure to reach a specified extrusion speed. After obtaining the correspondence between the air pressure and the extrusion speed, printing at different speed ratios of *v_sup_/v_ink_* could be realized by setting the printing platform moving speed *v_sup_* and the ink extrusion speed *v_ink_*.

**Figure A1:**
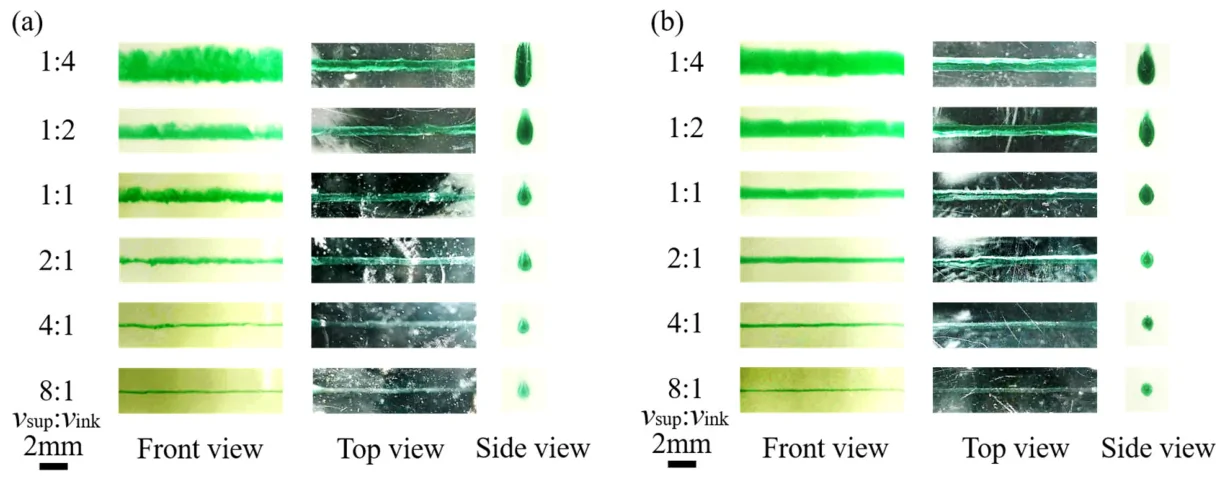
Phase diagrams of the printed filaments presented in three directions: front, top and side views at different speed ratios. (**a**) Ink-L and Sup-H; (**b**) Ink-M and Sup-H.

The processed images corresponding to the filaments in the three directions of front view, top view, and side view were obtained by photographing, as shown in Figure 3b and Figure A1. As can be seen from the front and top views, at *v_sup_/v_ink_* < 1, the ink was transitionally piled up in the restricted space and resulted in swollen filaments; at *v_sup_/v_ink_* > 1, the ink was dragged by the nozzle and obtained stretched filaments; and at *v_sup_/v_ink_* = 1, the printing resulted in a uniform filament with diameter equal to the nozzle inner diameter. This result was consistent with the previous study. The changes in filament diameter in the *z*-axis and *x*-axis directions were different. As seen in Figure 3c, the change in *d_z_* on the front view was greater than the change in *d_x_* on the top view throughout the variation in *v_sup_/v_ink_* from 0.25 to 8: *d_z_ > d_x_* at *v_sup_/v_ink_* < 1, *d_z_ < d_x_* at *v_sup_/v_ink_* > 1, and *d_z_ = d_x_* at *v_sup_/v_ink_* = 1. A transient gap was formed at the backward part of the nozzle when the moving nozzle sheared the support bath, and the transient gap was distributed along the *z*-axis plane. At *v_sup_/v_ink_* < 1, the ink filament with an excess volume fraction was more likely to grow upward along the transient gap, resulting in a “fin shape” filament, as shown in Figure 3d. At *v_sup_/v_ink_* > 1, the rapid movement of the nozzle stretched the filament, resulting in *d_z_ < d_x_*.

**Figure A2:**
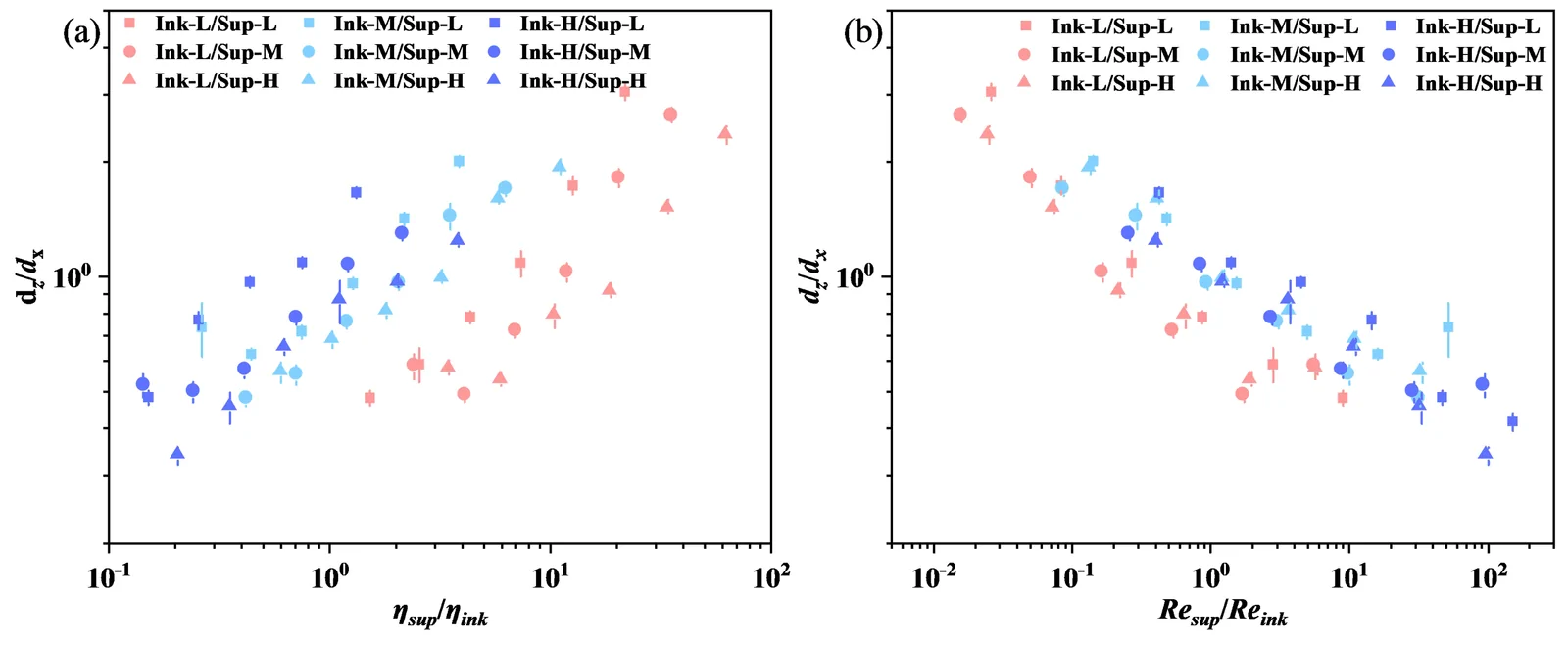
(**a**) Curve of *d_z_/d_x_* versus *η_sup_/η_ink_*; (**b**) curve of *d_z_/d_x_* versus *Re_sup_/Re_ink_*.

The filament diameter ratio *d_z_/d_x_* was an important parameter describing the roundness of the printed filament, with the ideal state of the *d_z_/d_x_* ratio being 1. However, the roundness of the printed filament would change with the printing parameters and rheological properties. As shown in Figure 3e and Figure A2 in the Appendix A, the *d_z_/d_x_* obtained from nine ink–support bath printing combinations at different speed ratios converged linearly in logarithmic coordinates. Spearman correlation analysis was used to obtain the correlation between the *v_sup_/v_ink_* and *d_z_/d_x_*. As shown in Table A2, the correlation rs = −0.935 between *v_sup_/v_ink_* and *d_z_/d_x_*, showing a high correlation, and the minus sign indicates a negative correlation. In addition, both the support baths and inks were shear-thinning fluids that exhibited different apparent viscosities at different shear rates, so their viscosity relationship may also have affected the morphology of the printed filaments during the printing process. Here, the local viscosities of the two fluids during the printing process were obtained from the viscosity curves by the local rates, as shown in Equations (3) and (4). The correlation rs = 0.687 between *η_sup_/η_ink_* and *d_z_/d_x_* at different viscosity ratios showed a moderate positive correlation. The Reynolds number describes the interaction between viscous and inertial forces during fluid motion, and the correlation rs = −0.890 between *Re_sup_/Re_ink_* and *d_z_/d_x_* showed a highly negative correlation. In addition, in the range of printing speed (0–20 mm/s), both *Re_sup_* and *Re_ink_* were less than 0.5, so their printing process followed the law of laminar flow, and the effect of viscous force was greater than that of inertia force. However, the change in viscosity and Reynolds number was caused by printing speed change, so *v_sup_/v_ink_* was the first significant factor in determining the filament shape.

**Figure A3:**
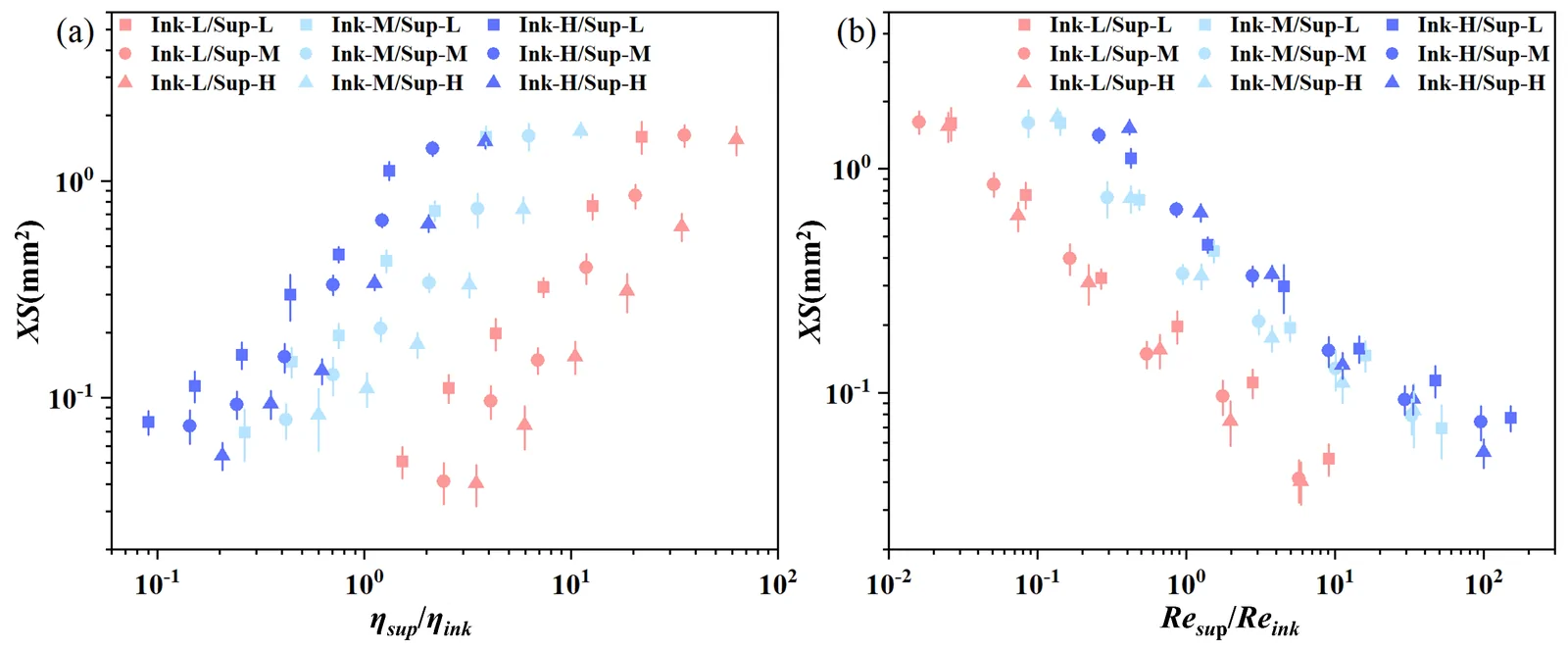
(**a**) Curve of *XS* versus *η_sup_/η_ink_*; (**b**) curve of *XS* versus *Re_sup_/Re_ink_*.

Since the image captured in the side view was a side projection of the filament, it was not representative of the average cross-section of the filament. Therefore, the average cross-sectional area of the filament *XS* was calculated using the average of *d_z_* and *d_x_*, as shown in Equation (5). And it was applied to evaluate the size of the cross-sectional shape of the filament. As shown in Figure 3f and Figure A3 and Table A2 in the Appendix A, the *XS* obtained from nine ink–support bath printing combinations at different speed ratios converge linearly in logarithmic coordinates, and the correlation rs = −0.983 between *v_sup_/v_ink_* and *XS* shows a highly negative correlation. *η_sup_/η_ink_* showed a moderate positive correlation with *XS* with rs = 0.586. *Re_sup_/Re_ink_* showed a strong negative correlation with *XS* with rs = −0.856. The correlation results were similar to those of the filament diameter ratio, indicating the decisive role of *v_sup_/v_ink_* on the volumetric morphology of the filaments. In summary, *d_z_ = d_x_* = d at *v_sup_/v_ink_* = 1 when the shape of the filament was more rounded and the size of the filament cross-section was closer to the cross-sectional area of the nozzle.

(3) $${\dot{\gamma }}_{sup}=\frac{v_{sup}}{D}$$

(4) $${\dot{\gamma }}_{ink}=\frac{8v_{ink}}{d}$$

(5) $$XS=\frac{1}{4}\pi d_{z}d_{x}$$

#### 2.2.2. Contour Quality of Deposited Filaments

**Figure 4:**
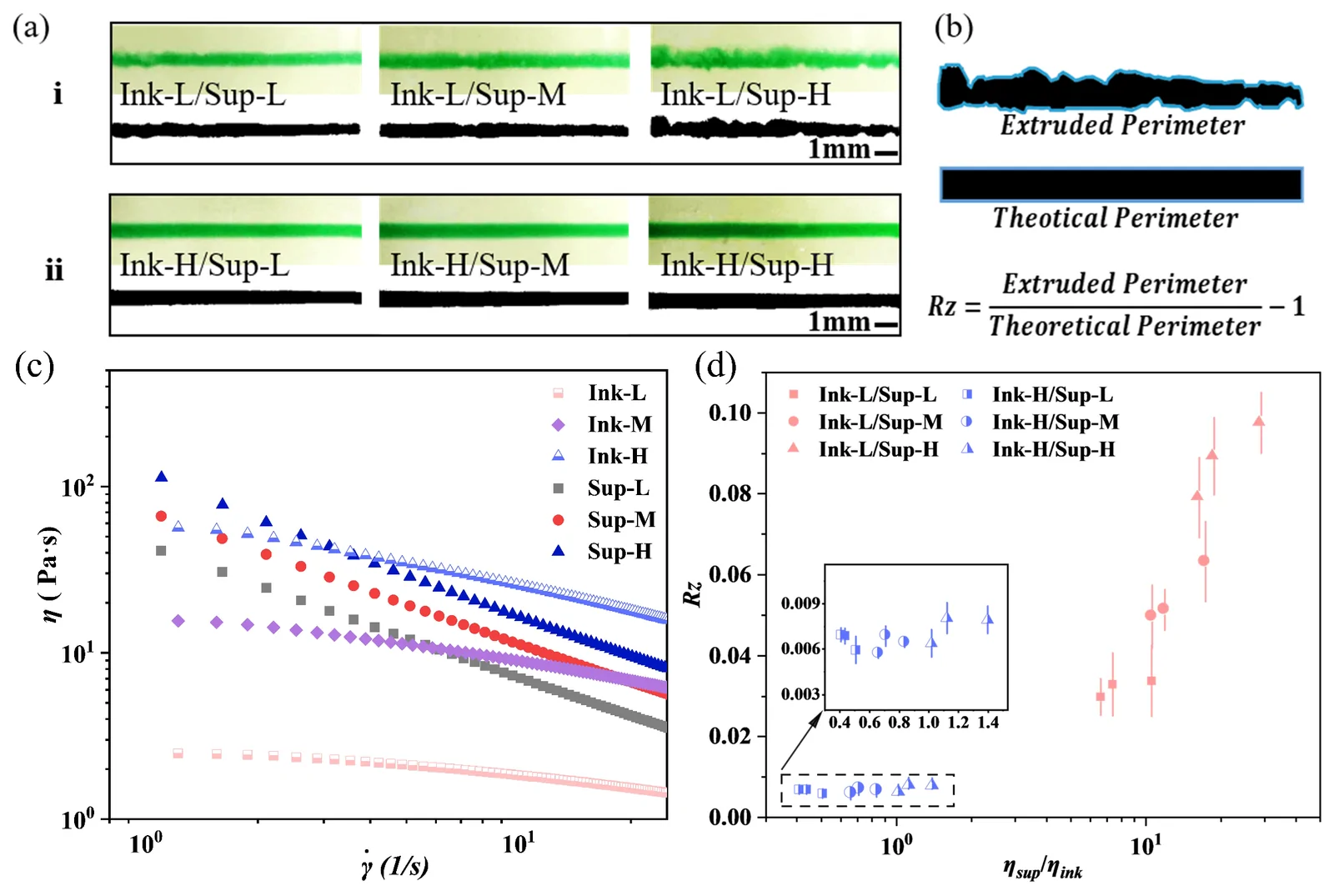
(**a**) Processed images of filaments at *v_sup_/v_ink_* = 1 with corresponding binarized processed images: (**i**): processed images obtained from the printing of low-viscosity ink Ink-L; (**ii**): processed images obtained from the printing of high-viscosity ink Ink-H. (**b**) Definition of the contour irregularity *CI* of the filament. (**c**) Shear rate and viscosity relationship curves for inks and support baths. (**d**) Variation in filament contour irregularity index *CI* versus viscosity ratio *η_sup_/η_ink_* for *v_sup_/v_ink_* = 1 (*v_ink_* = 3.1 mm/s). η is the viscosity and $\dot{\gamma }$ is the shear rate.

The *d_z_/d_x_* values obtained from different inks printed in different support baths were similar at the same speed ratio, indicating comparable volumetric morphology, whereas their contour regularity differed markedly. The experimental images revealed obvious differences in filament contour regularity among different ink–support bath combinations. The difference was particularly evident along the side-view filament boundary associated with *d_z_*, which was therefore selected for representative analysis. The processed images of the filaments obtained by printing Ink-L and Ink-H in Sup-L, Sup-M, and Sup-H and their black-and-white images obtained by ImageJ (1.53t) are shown in Figure 4a. As seen in Figure 4a(i,ii), filaments printed with Ink-L exhibit visibly wavy and irregular contours, whereas those printed with Ink-H show smoother and more regular boundaries. The contour regularity of the printed filament was quantified using an image-based contour irregularity index (*CI*), as shown in Figure 4b.

To place the rheological characterization in the context of actual printing, the local shear rates experienced by the ink and the support bath during deposition can be estimated from the printing parameters. For the ink inside the nozzle, the wall shear rate is approximately ${\dot{\gamma }}_{ink}\approx {8v}_{ink}/d$, where d=0.6 mm is the nozzle inner diameter. For the support bath around the moving nozzle, the shear rate is approximately ${\dot{\gamma }}_{sup}\approx v_{sup}/D$, where D=0.9 mm is the nozzle outer diameter. With the typical $v_{ink}$ values of 2–4 mm/s and $v_{sup}$ values of 0.5–32.8 mm/s used in this study, the ink experiences ${\dot{\gamma }}_{ink}\approx 26.7–53.3$ s^−1^ inside the nozzle, while the support bath experiences ${\dot{\gamma }}_{sup}\approx 0.67–36.4$ s^−1^ around the moving nozzle. These ranges fall within the shear rate window shown in Figure 4c, confirming that the rheological measurements cover the relevant deformation rates during printing. It can be seen from Figure 4c that at the same shear rate, the viscosity of Ink-L is always lower than that of the three support baths, and the viscosity of Ink-H is higher than that of the three support baths; thus, for the support baths, Ink-L belongs to low-viscosity ink, and Ink-H belongs to high-viscosity ink.

As shown in Figure 4d, for the low-viscosity ink Ink-L, the *CI* of the filament increased as *η_sup_/η_ink_* increased. This is because the low-viscosity ink, with relatively weak viscous resistance, was more likely to penetrate into and redistribute within the transient gap during bath healing, thereby producing more irregular filament contours. For the high-viscosity ink Ink-H, as *η_sup_/η_ink_* increased, *CI* remained within a narrow range of 0.005–0.009, indicating that the contour irregularity of filaments printed with high-viscosity ink was only weakly affected by *η_sup_/η_ink_*. The higher viscous resistance of the ink hindered penetration and redistribution into the transient gap during support bath healing, thereby preserving a smoother and more regular filament contour. Therefore, printing a high-viscosity ink in a relatively low-viscosity support bath was more likely to yield filaments with lower contour irregularity. This trend is consistent with the interfacial stability analysis of Friedrich and Seppala [26,27], who showed that excessive interfacial stress destabilizes the filament boundary and promotes defects. In the present system, the higher viscous resistance of Ink-H plays a similar stabilizing role by hindering penetration into the transient gap during bath healing, which explains why *CI* remains within 0.005–0.009 even when the bath viscosity varies.

#### 2.2.3. Deposition Position of the Filament on the z-Axis

**Figure 5:**
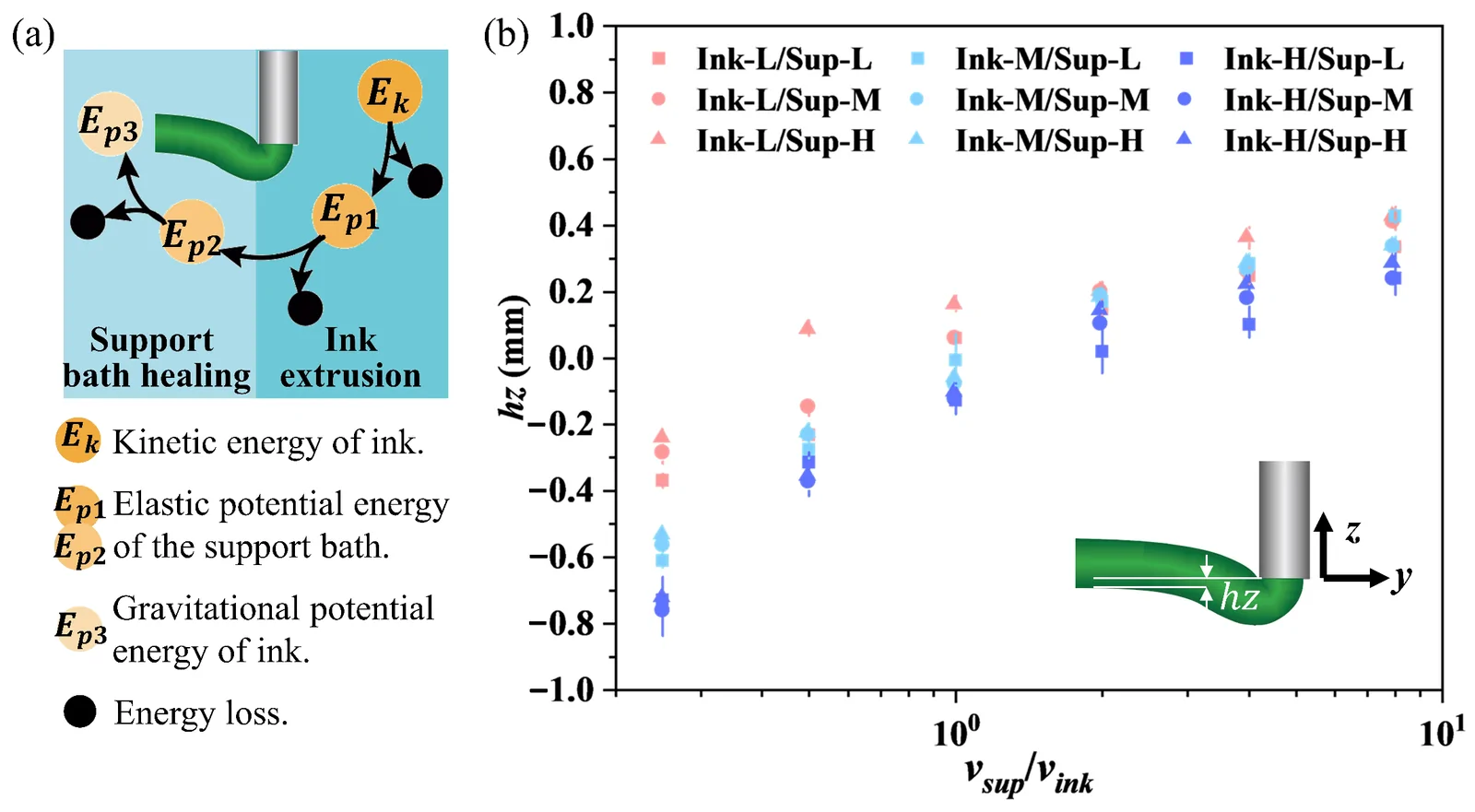
(**a**) Schematic diagram of the energy conversion relationship of the printing process. (**b**) Curve of *hz* versus *v_sup_/v_ink_* (*v_ink_* = 2 mm/s). Here, *hz* is the deposition position of the filament along the *z*-axis relative to the nozzle-tip plane (positive when the filament is above the plane, negative when below).

The deposition position of the filament on the *z*-axis *hz* reflects the positional accuracy of the embedded printing. Ideally, the deposition position of the filament is directly below the nozzle. As illustrated in Figure 5a, there was kinetic energy when the ink was extruded during the printing process, and this kinetic energy caused a squeezing effect on the support bath under the nozzle. Since the support bath was a viscoelastic fluid with an elastic modulus [40], this kinetic energy was partly converted into the elastic potential energy of the support bath and partly dissipated. When the nozzle moved forward, the support bath healed, the elastic potential energy was released and partially converted into the gravitational potential energy of the deposited filaments, which caused the filament to be displaced in the *z*-axis. In this paper, *hz* was used to measure the final deposition position of the filament. When the lower edge of the filament was below the plane of the nozzle tip, *hz* was negative, and vice versa, *hz* was positive.

**Figure A4:**
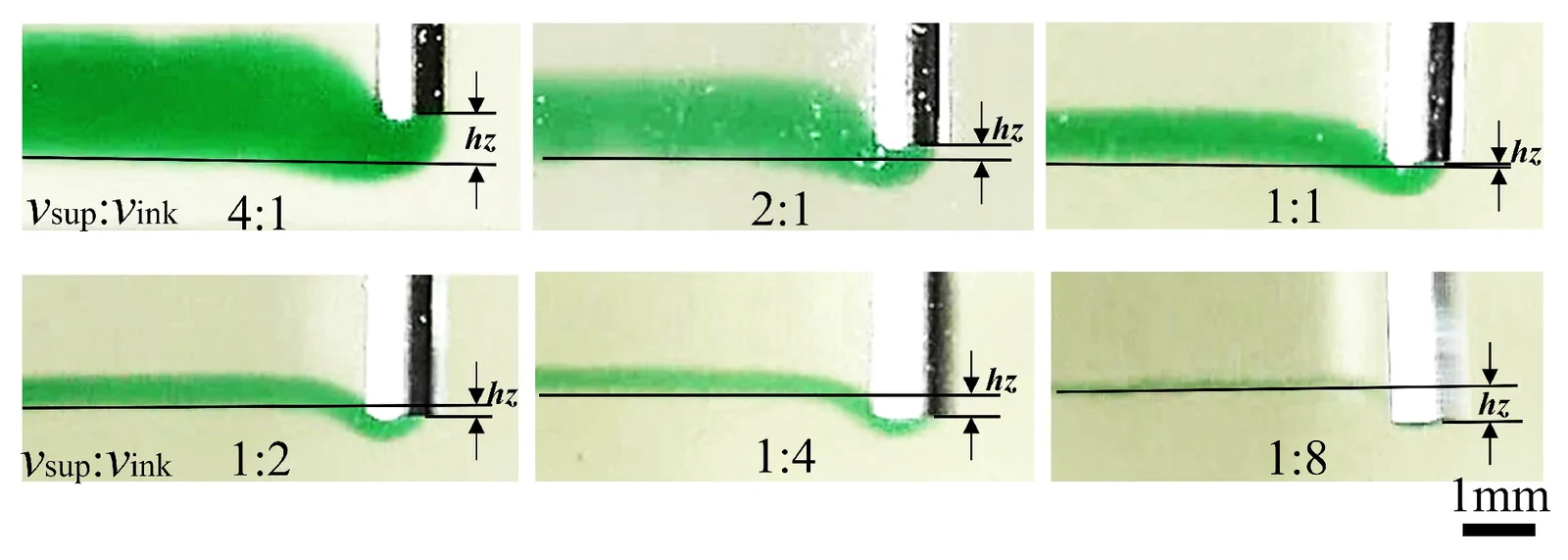
Phase diagrams of the deposition positions of the filaments at different speed ratios.

**Figure A5:**
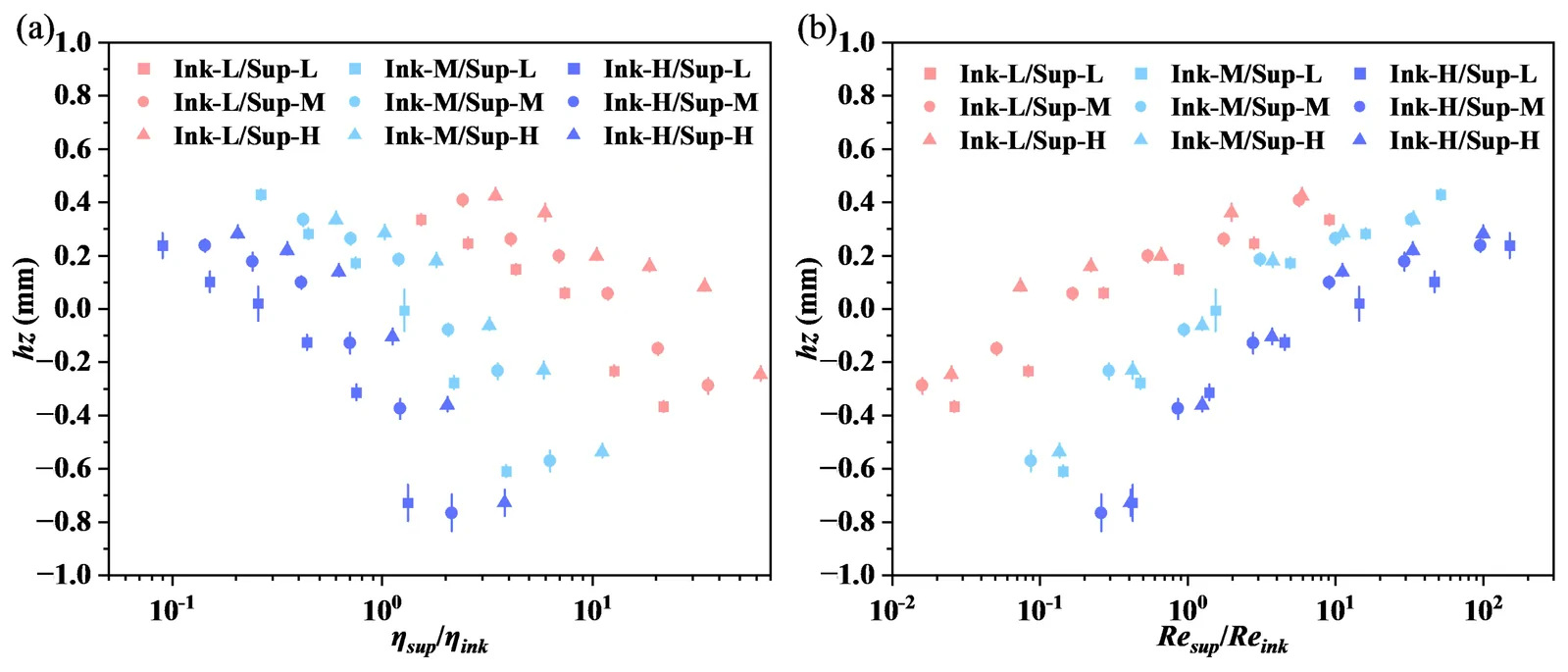
(**a**) Curve of *hz* versus *η_sup_/η_ink_*; (**b**) curve of *hz* versus *Re_sup_/Re_ink_*.

As shown in Figure 5b and Figure A4 in the Appendix A, as *v_sup_/v_ink_* increased, *hz* gradually increased from negative to positive values and the filament position moved upward along the *z*-axis direction. From the previous sections, it is known that the volume of ink deposited per unit length was inversely proportional to *v_sup_/v_ink_*. If *v_sup_* increased while *v_ink_* remained constant, the volume fraction of ink deposited per unit length decreased, and then *hz* increased. In addition, the *hz* of ink Ink-H was smaller than the other groups at the same speed ratio. Because Ink-H had the highest viscosity among the three inks, and it also had the highest energy storage modulus, so it could dissipate more elastic potential energy released from the support bath during the support bath healing process, which resulted in a smaller *hz* compared with other inks. Among the three groups of support baths, the *hz* of the ink filaments printed in Sup-H tended to be the largest under the same speed ratio. Because Sup-H had the largest content of microgel particles and highest yield stress among the three groups of support baths, its ability to convert and store elastic potential energy under the same kinetic impact was also the strongest, and the elastic potential energy released after the nozzle moved forward the bath was also the highest. As shown in Table A2 and Figure A5 in the Appendix A, the Spearman correlation rs = 0.949 between *hz* and *v_sup_/v_ink_* showed a highly positive correlation; the correlation rs = −0.375 between *hz* and *η_sup_/η_ink_* showed a weak negative correlation; and the correlation rs = 0.713 between *hz* and *Re_sup_/Re_ink_* showed a strong positive correlation. The results showed that the *hz* was related to the *v_sup_/v_ink_* and the viscoelastic properties of the material, in which *v_sup_/v_ink_* was the dominant factor, and *hz* was close to zero when *v_sup_/v_ink_* = 1.

### 2.3. Diffusion of Ink Filaments in the Support Bath

**Figure 6:**
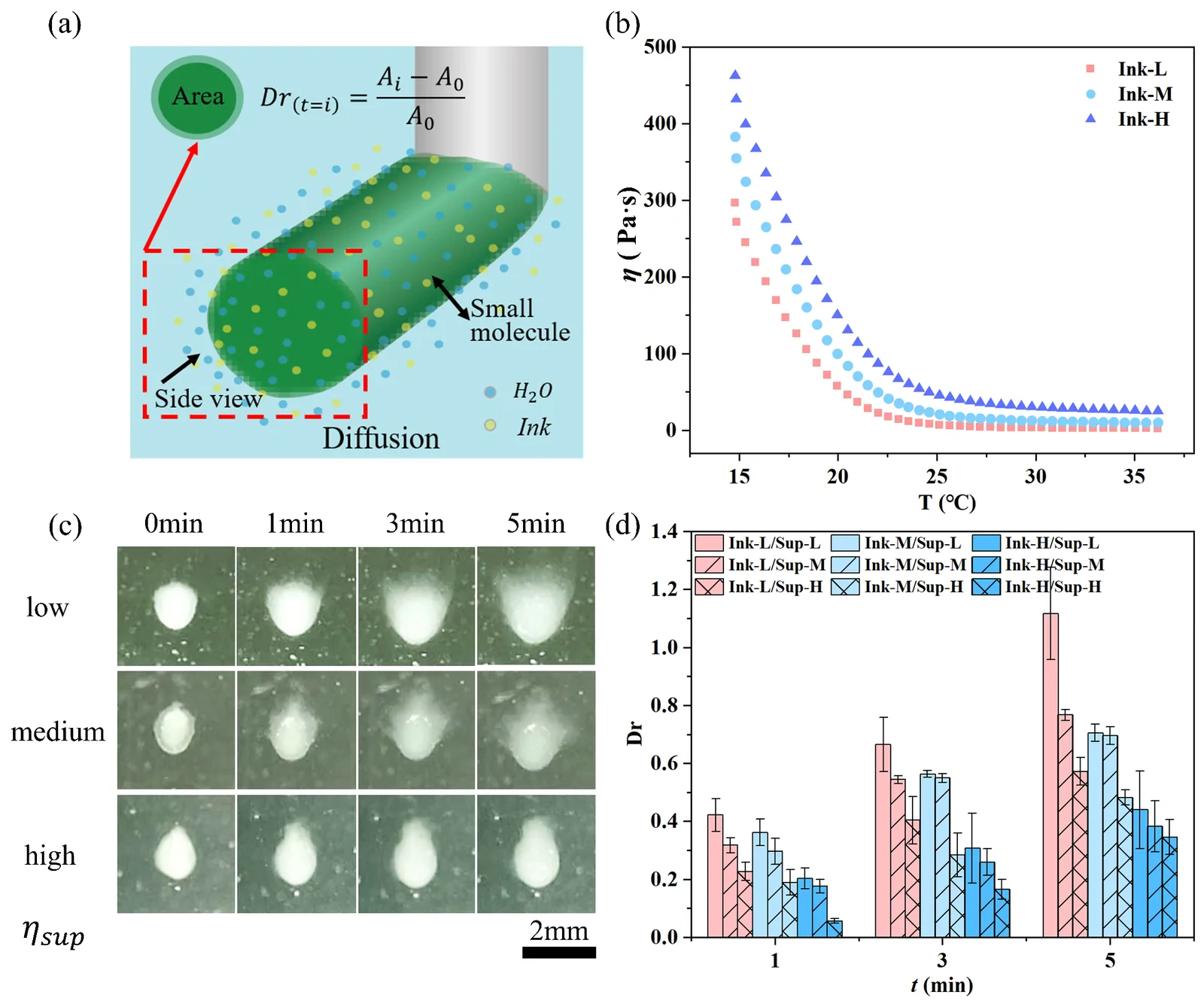
(**a**) Schematic diagram of diffusion changes and characterization indexes. (**b**) The complex viscosity of ink versus scanning temperature curve (scanning speed 5 °C/min). (**c**) Phase diagram of the filament change with time in the support bath (Ink-H, *v_ink_* = 2 mm/s). (**d**) Diffusion ratio *Dr* versus t for different ink–support bath combinations. Here, *Dr* is the diffusion ratio, *Ai* is the side-view projected area of the filament at i minutes after printing, and *A_0_* is the initial area at the moment of printing.

The previous sections described the effects of printing process parameters and material properties on the short-term morphology of the embedded printing process. A good short-term morphology is the foundation for achieving the desired structure in embedded printing. However, the morphology of the ink filaments will change in the support bath during the cross-linking process, and diffusion is the dominant factor. As shown in Figure 6a, in the diffusion system, the ink was the high-concentration diffusion source. The small gelatin and sodium alginate molecules in the ink diffused into the support bath, and the water molecules in the support bath infiltrated into the ink filaments. To characterize the degree of diffusion, the diffusion ratio *Dr* was used to measure the degree of diffusion at different times.

**Figure A6:**
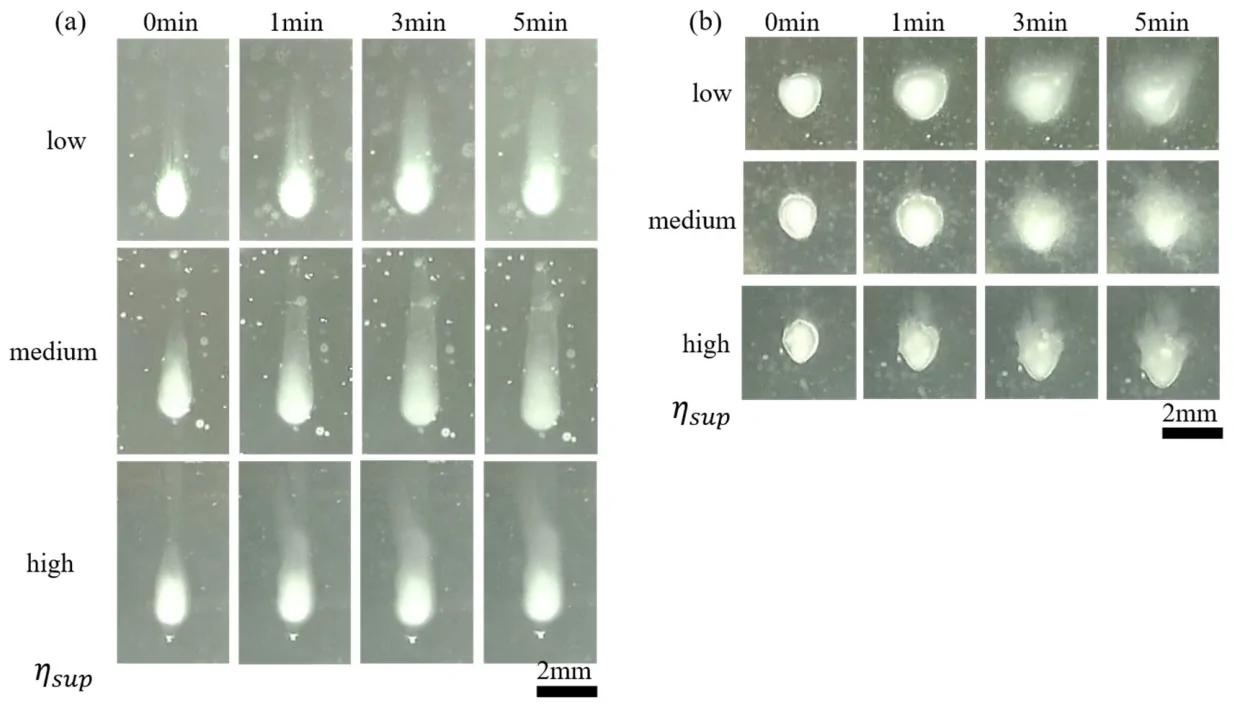
Phase diagram of filaments in the support bath over time at the end of printing. (**a**) Ink-L, *v_ink_* = 2 mm/s; (**b**) Ink-M, *v_ink_* = 2 mm/s.

The diffusion ratio *Dr* was highly related to the degree of cross-linking of the ink filament. Since the gelatin in the ink was a thermosetting material, the properties of the ink at different temperatures were studied. The ink was subjected to a temperature scanning rheology test from 37 °C to 15 °C at a scanning speed of 5 °C/min. As shown in Figure 6b, when the temperature of the ink was reduced to 25 °C, the viscosity began to change dramatically, indicating that the ink began to cross-link at this temperature, turning from sol to gel. Thus, the temperature of the support bath was set at 25 °C to ensure that the ink filaments could undergo the cross-linking reaction right after being printed. The side-view processed images of the ink filament at 0 min, 1 min, 3 min, and 5 min after printing were obtained, as shown in Figure 6c and Figure A6 in the Appendix A. As the resting time increased, the area of ink filaments on the side view increased. There was diffusion of ink filaments, and the ink filaments exhibited different change rates in different viscosity combinations of the inks and the support baths. To quantify the extent of their changes, the diffusion ratio *Dr* was characterized and is shown in Figure 6d. It can be seen that *Dr* increased with the increase in t. The mean value of *Dr*(t = 1) for the nine printing combinations was about 0.2, the mean value of *Dr*(t = 3) was about 0.4, and the mean value of *Dr*(t = 5) was about 0.5. In addition, the *Dr* of Ink-H was smaller relative to Ink-M and Ink-L, and the *Dr* of Sup-H was smaller relative to Sup-L and Sup-M. for the Ink-H and Sup-H combination, the *Dr* was 0.056, 0.16, and 0.34 for t = 1, 3, 5 min, while for the Ink-L and Sup-L combination, the *Dr* was 0.42, 0.66, and 1.12 for t = 1, 3, 5 min. The high-viscosity combination could reduce about 70% of the diffusion. According to the Stokes–Einstein equation [41], the higher the viscosity, the lower the diffusion coefficient; thus the Ink-H and Sup-H with higher viscosity had lower diffusion. Moreover, a high-viscosity support bath means that there were more Carbomer microgel particles and there was less free water in the support bath, which also slowed down diffusion. The suppression of diffusion by high-viscosity combinations is consistent with the Stokes–Einstein relation [41] and with Lei et al. [31], who reported 10–20% dimensional deviation caused by diffusion in hydrophilic systems; the present *Dr* quantifies this early-stage evolution directly. In summary, when printing hydrophilic inks in a hydrophilic bath, diffusion is unavoidable and can be slowed down by appropriately increasing the viscosity of the ink and the support bath.

### 2.4. Printing of 3D Structures

**Figure 7:**
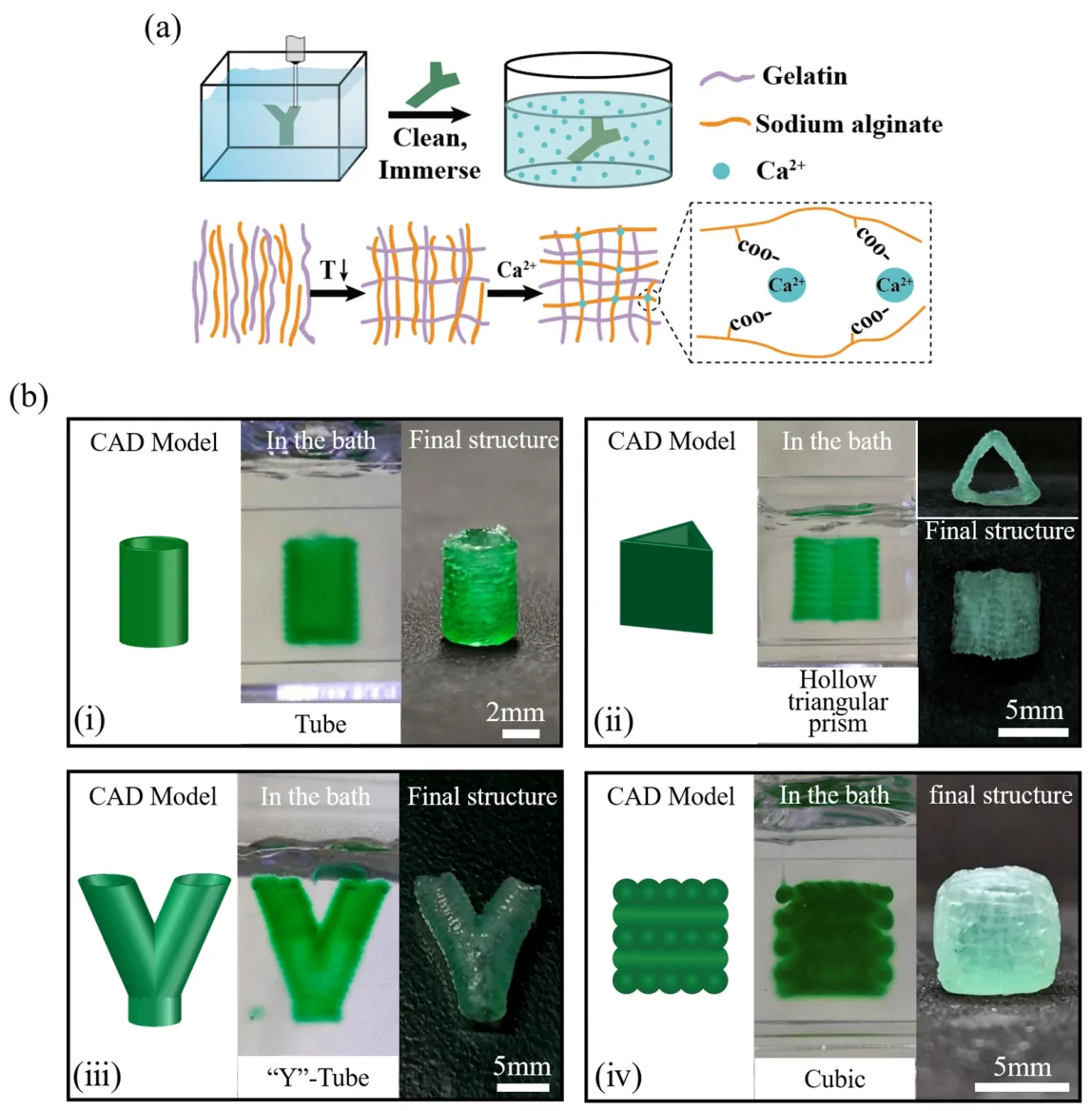
(**a**) Schematic illustration of the cross-linking mechanism of the ink material. (**b**) Computer-aided design (CAD) models and the corresponding embedded three-dimensional printed structures: (**i**) circular tube; (**ii**) hollow triangular prism; (**iii**) Y-shaped circular tube; (**iv**) cube.

Based on the results obtained in the previous section, the printing speed ratio of the recommended filament pattern (*v_sup_/v_ink_* = 1) was determined, as well as the printing layer height *h* = *d_z_* + *hz* = 0.6 mm. Ink-H was used to print three-dimensional constructions in Sup-H, and *v_sup_* = *v_ink_* = 4.1 mm/s. The nozzle inner diameter was 0.6 mm. The cross-linking process of the ink is shown in Figure 7a; the printed structure was left at room temperature for 5 min at 25 °C until the gelatin cooled down and gelatin cross-linking occurred to maintain the structure’s morphology. Subsequently, the structure was exported from the support bath and cleaned, and then immersed in CaCl_2_ solution for 30 min to perform the second step of cross-linking of sodium alginate with calcium ions. Finally, the final hydrogel structure was obtained, as shown in Figure 7b. The dimensional expansion ratios of the four structures are listed in Table A3. These results demonstrate that the identified parameter window can support the fabrication of multiple hydrogel geometries. However, the fidelity depended on structural geometry: tubular and continuous-path structures showed better shape retention, whereas the cubic structure displayed more obvious deformation at corners and edges. This was likely associated with local material accumulation at turning regions, support bath relaxation/healing effects, and continued filament evolution before full secondary cross-linking. Accordingly, the present process window is more suitable for continuous filament trajectories and tubular structures than for geometries with sharp corners or more complex unsupported features. The comparison of the present work with representative embedded printing studies is shown in Table 2 from the evaluation coverage and key conclusion. This work not only studies the filament printing process, but also the early evolution of the filament in the supporting bath, which also affects the final shape of the filament.

## 3. Conclusions

This study developed an integrated engineering evaluation route for analyzing filament formation and early-stage evolution during embedded printing of gelatin/sodium alginate hydrogels in Carbomer support baths. By combining cross-sectional geometry (*d_z_/d_x_*, *XS*), image-based contour irregularity (*CI*), deposition position (*hz*), and diffusion ratio (*Dr*), the work establishes a practical process window for a representative hydrophilic ink–hydrophilic bath system. The results indicate that the printing speed ratio *v_sup_/v_ink_* is the dominant factor controlling filament geometry and deposition position. When *v_sup_/v_ink_* approaches 1, the printed filaments exhibit more balanced cross-sectional morphology and improved positional consistency. In addition, ink viscosity mainly affects contour quality and short-term diffusional stability: higher-viscosity inks maintain contour irregularity within a narrow range of 0.005–0.009, and the high viscosity and support bath combination slows diffusional evolution by about 70%. These findings suggest that stable embedded printing depends on coordinated control of deposition kinematics and rheological matching, and rheological matching also controls the diffusion process. While many studies focus on filament formation during the printing process, this study suggests that the diffusion after printing is also important for the final shape of the printed filament and should be considered when matching the materials’ rheology. Future studies could focus on how the cells infect the printing kinetics and diffusion process. While this study only included hydrophilic embedded printing, more material combinations, such as hydrophilic and hydrophobic, could be studied in the future.

## 4. Materials and Methods

### 4.1. Preparation of Materials

Support bath preparation: Carbomer (2020, Macklin, Shanghai, China) was used as the support bath material for all the printing experiments. The Carbomer powder was weighed and dispersed in deionized water to prepare 0.2 wt.%, 0.4 wt.% and 0.8 wt.% Carbomer dispersions, and after the Carbomer particles were thoroughly hydrated, the pH of the Carbomer dispersion was adjusted to 7.0 ± 0.2 by adding an appropriate amount of 5 mol/L sodium hydroxide solution (Sinopharm Chemical Reagent, Shanghai, China). Sup-L, Sup-M, and Sup-H Carbomer support baths were obtained, as shown in Table 3.

Ink preparation: Ink-L, Ink-M, and Ink-H inks were prepared by adding 1 wt.%, 2 wt.%, and 3 wt.% of sodium alginate (Krohne, Chengdu, China) to 10 wt.% of gelatin (Aladdin, gel strength ~250 g Bloom, Shanghai, China) and deionized water, as shown in Table 3. The inks were prepared by stirring sodium alginate and gelatin in deionized water continuously for 4–6 h at 37 °C in a water bath. To enhance the visualization of the inks during the printing process, 0.5 wt.% of dark green edible color was added during the ink preparation.

Carbomer removal solution: The 0.9 wt.% sodium chloride solution was prepared by weighing appropriate amounts of sodium chloride crystals (Sinopharm Chemical Reagent, Shanghai, China) and dissolving them in deionized water with stirring. Carbomer micro-hydrogel can be removed from the printed structure by immersion and washing with sodium chloride solution.

Sodium alginate cross-linking solution: The 3 wt.% calcium chloride solution was prepared by weighing an appropriate amount of calcium chloride crystals (Sinopharm Chemical Reagent, Shanghai, China) and dissolving them in deionized water with stirring. The printed structure was immersed in the calcium chloride solution to form a calcium alginate hydrogel by the cross-linking reaction of alginate and calcium ions.

### 4.2. Rheological Testing

The rheological properties of Carbomer micro-hydrogels and inks were measured using a rotational rheometer (HR 20, TA Instruments-Waters LLC, New Castle, DE, USA) with 25 mm diameter parallel plates with a gap size of 1 mm, and the shear rate was varied from 0.01 to 100 s^−1^ to measure the dynamic viscosity of the materials and shear stresses, where the Carbomer was tested at 25 °C and the ink was tested at 37 °C. In addition, the complex viscosity of the ink was measured by oscillating at 0.1% strain and 1.5 Hz frequency, and the instrument was cooled from 37 °C to 15 °C at a rate of 5 °C/min for temperature scanning oscillatory tests.

### 4.3. Printing Device

**Figure 8:**
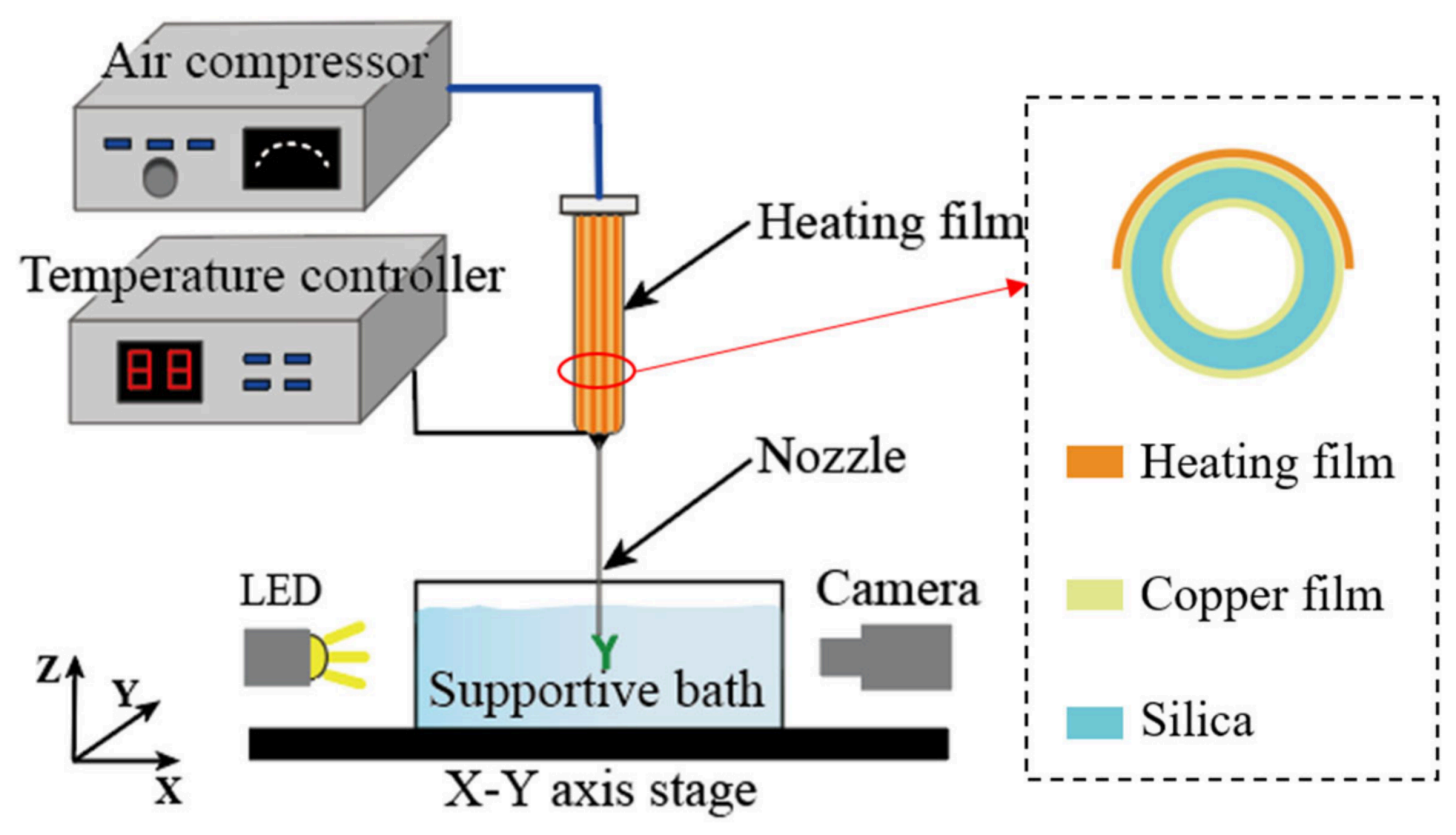
Schematic diagram of the printing device.

The printing device contains a moving platform, an air supply device, a heating and holding device, and a filming device, as shown in Figure 8. The moving platform was based on the traditional 3D printing *xyz*-axis moving platform; the liquid reservoir tube was fixed on the *z*-axis together with the temperature control unit, while the support bath was placed on the *xy*-axis platform, which moved at a speed of 0–200 mm/s. The liquid reservoir tube was loaded with ink, and the upper port was connected to the air supply device. The air pressure was supplied from an air compressor (TW19-90S, Shanghai Tuowen Machinery Co., Ltd., Shanghai, China). The temperature control unit consisted of a PI heating film (70 × 20 mm, 19.2Ω), a copper film, a thermally conductive silica, a temperature sensor, and a temperature controller (AI-208D2L, Xiamen Yudian Automation Technology Co., Ltd., Xiamen, China). The temperature control unit maintained the temperature of the ink at 37± 0.5 °C. The filming device consisted of a microscope (Dino-Lite VIDY Optical (WuXi) Co., Ltd., Wuxi, China) and an LED light source. The ink extrusion speed was obtained from the mass fluid rate, as shown in Equation (6).

(6) $$v_{ink}=\frac{4Q_{m}}{\rho \pi d^{2}}$$

Here, *Q_m_* is the mass flow rate of the ink obtained by the weighing method, ρ is the ink density, and *d* is the inner diameter of the nozzle (0.6 mm).

### 4.4. Characterization Parameters and Methods

The video of the printing process was captured for each print, with the filament morphology captured in the order of the front-view Y-Z plane, the side-view X–Z plane, and the top view X-Y plane. The acquisition time of each plane was about 5 s, and the acquisition could be completed within 30 s, ×50 with the Dino-Lite microscope. The image of the filament position pattern was taken from the front-view Y-Z plane. Morphological images of the cross-linking evolution process were taken from the side-view X-Z plane, and the video was captured from t = 0 min to t = 5 min after printing. Subsequently, the images were intercepted from the video, and to avoid the effect of air pressure instability at the beginning and end of the print, the measurements were taken from the middle part of the filament, and the length taken was two-thirds of the overall length. All experiments were performed at least five times. The images were binarized and measured using MATLAB R2023b and ImageJ 1.53t. The contour irregularity *CI* was calculated by Equation (7). The diffusion ratio *Dr* was calculated by Equation (8).

(7) $$CI=\frac{P_{e}}{P_{th}}-1$$

(8) $$Dr_{\left(t=i\right)}=\frac{A_{i}-A_{0}}{A_{0}}$$

where *Pe* was the perimeter of the filament image, *Pth* was the perimeter of the rectangle drawn from length and average diameter of the filament segment, *A_i_* was the area at *i* minutes after the printing, and *A_0_* was the initial area at the moment of printing.

### 4.5. 3D Structure Printing and Cross-Linking

The three-dimensional models (tube, hollow triangular prism, “Y”-tube, and cube) were created in computer-aided design (CAD)2019. The printing was performed on a commercial extrusion-based embedded printing device manufactured by Yijie (China). The device can accomplish printing path planning via multi-layer configuration in CAD 2019 software. The cross-linking reaction of the ink material was divided into two steps: The first step was thermal cross-linking of gelatin, where the temperature of the ink decreased from 37 °C to room temperature at 25 °C, during which the cross-linking reaction occurred and the printed structure was initially maintained. The second step was to immerse the structure (after being exported from the support bath and cleaned) in the 3 wt.% CaCl_2_ solution for 30 min to allow cross-linking of the alginate with the calcium ions. After the two-step cross-linking reaction, gelatin/calcium alginate hydrogels with double cross-linked network interpenetrating structures could be prepared.

## Figures and Tables

**Table 1:** Rheological coefficients of support baths and inks.

	$\sigma_{0}$	*K*	*n*
Sup-L	14.21 ± 1.525	27.77 ± 1.146	0.31 ± 0.006
Sup-M	29.61 ± 1.170	39.67 ± 0.858	0.32 ± 0.003
Sup-H	87.98 ± 0.734	28.69 ± 0.437	0.43 ± 0.003
Ink-L	N/A	4.37 ± 0.035	0.65 ± 0.002
Ink-M	N/A	30.34 ± 0.343	0.50 ± 0.003
Ink-H	N/A	103.01 ± 1.018	0.41 ± 0.002

**Table 2:** Comparison of the present work with representative embedded printing studies.

Study	Evaluation Coverage	Key Conclusion
Jin et al. [22]	Deposition only: filament diameter	Speed ratio and storage-modulus ratio govern filament printability
Hua et al. [23]	Deposition only: filament formation taxonomy	Thixotropic time and yield stress jointly govern filament quality and allowable path speed
Grosskopf et al. [25]	Bath rheology, shape retention	Yield stress, viscoelasticity and Oldroyd number control yielded-region dimensions and print fidelity
Friedrich & Seppala [26,27]	Deposition only: cross-section and defects	Interfacial stress governs contour regularity
Lei et al. [31]	Post-deposition only: diffusion and shapeability	In-bath diffusion causes dimensional deviation
This work	Deposition, early evolution (*d_z_/d_x_*, *XS*, *CI*, *hz*, *Dr*)	Integrated evaluation route; speed-ratio-dominant; diffusion reduction

**Table 3:** Classification of the ratio of support baths and inks.

Category	Name	Material Ratio
Support bath	Sup-L	0.2 wt.% Carbomer
	Sup-M	0.4 wt.% Carbomer
	Sup-H	0.8 wt.% Carbomer
Ink	Ink-L	1 wt.% Sodium Alginate + 10 wt.% Gelatin
	Ink-M	2 wt.% Sodium Alginate + 10 wt.% Gelatin
	Ink-H	3 wt.% Sodium Alginate + 10 wt.% Gelatin

## Data Availability

The data presented in this study are available on request from the corresponding author.

## Appendix A

**Table A1:** Rheological coefficients of inks (low-shear region, for $\dot{\gamma }$ lower than 10).

	$\sigma_{0}$	*K*	*n*
Ink-L (low-shear region)	N/A	2.22 ± 0.112	0.96 ± 0.022
Ink-M (low-shear region)	N/A	15.38 ± 0.710	0.80 ± 0.020
Ink-H (low-shear region)	N/A	62.10 ± 1.778	0.63 ± 0.012

**Table A2:** Table of Spearman correlation coefficients for dimensionless parameters.

Metrics		Spearman Coeff rs	Spearman P
*d_z_/d_x_*	*v_sup_/v_ink_*	−0.935 **	3.91 × 10^−25^
	*η_sup_/η_ink_*	0.687 **	9.48 × 10^−9^
	*Re_sup_/Re_ink_*	−0.890 **	2.23 × 10^−19^
*XS*	*v_sup_/v_ink_*	−0.983 **	3.20 × 10^−40^
	*η_sup_/η_ink_*	0.586 **	3.00 × 10^−6^
	*Re_sup_/Re_ink_*	−0.856 **	1.70 × 10^−16^
*hz*	*v_sup_/v_ink_*	0.949 **	1.17 × 10^−27^
	*η_sup_/η_ink_*	−0.375 **	0.005
	*Re_sup_/Re_ink_*	0.713 **	1.42 × 10^−9^

(|rs| > 0.9 highly correlated; 0.7–0.9 strongly correlated; 0.4–0.7 moderately correlated; 0.2–0.4 weakly correlated; <0.2 very weak/no correlation) (** denotes *p* < 0.01).

**Table A3:** Designed and final measured dimensions of various printed structures.

Structure	Circular Tube	Hollow Triangular Prism	Y-Shaped Circular Tube	Cube
Target diameter/side length	4 mm	5 mm	4 mm	6 mm
Target height	6 mm	6 mm	15 mm	6 mm
Measured diameter/side length	4.8 ± 0.3 mm	5.6 ± 0.3 mm	4.7 ± 0.3 mm	6.5 ± 0.3 mm
Measured height	6.5 ± 0.3 mm	6.2 ± 0.3 mm	15.3 ± 0.3 mm	6.5 ± 0.3 mm
Relative deviation in diameter/side length	+20%	+12	+17.5%	+8.3%
Relative deviation in height	+8.3%	+3.3%	+2%	+8.3%
